# Supplementary material for: A Likelihood Approach for Real-Time Calibration of Stochastic Compartmental Epidemic Models
Source: PLoS Comput Biol. 2017 Jan 17;13(1):e1005257. doi: 10.1371/journal.pcbi.1005257 (PMC5240920; doi:10.1371/journal.pcbi.1005257)
Supplement: S1 File — (TAR.GZ) [file pcbi.1005257.s014.tar.gz › HSPH_Online-SI-Revision/EnKF07/n10-severe/n10-severe_EnKF2_table-8.pdf]

1	newly 3 weeks spec	291.	203.92	{3., 765.}	116.289	1.07621	newly 3 weeks cumul	664.	1236.72	{161., 4141.}	182.494	0.548451	newly 5 weeks spec	473.	57.2	{0., 54.}	94.8922	2.12879	newly 5 weeks cumul	1512.	1389.92	{176., 5344.}	122.208	0.75096	newly 59 weeks cumul	6569.	1561.52	{181., 7217.}	82.7657	1.16778
2	newly 3 weeks spec	216.	27.27	{0., 71.}	87.662	1.64411	newly 3 weeks cumul	425.	510.44	{151., 919.}	67.8259	0.293001	newly 5 weeks spec	426.	6.86	{0., 13.}	98.3897	2.71214	newly 5 weeks cumul	1146.	529.54	{177., 919.}	59.8883	0.480755	newly 61 weeks cumul	6678.	541.5	{179., 919.}	91.8913	1.21435
3	newly 3 weeks spec	232.	255.87	{0., 708.}	112.944	1.19846	newly 3 weeks cumul	460.	3747.14	{739., 8965.}	720.235	0.768342	newly 5 weeks spec	491.	73.72	{0., 249.}	88.7373	2.10608	newly 5 weeks cumul	1296.	3951.1	{752., 10 215.}	223.884	0.450277	newly 63 weeks cumul	5504.	4108.39	{752., 10 601.}	61.5827	0.410761
4	newly 3 weeks spec	222.	111.73	{21., 245.}	79.527	0.659037	newly 3 weeks cumul	397.	614.32	{254., 1214.}	87.33	0.287872	newly 5 weeks spec	435.	46.	{2., 104.}	90.5931	1.47408	newly 5 weeks cumul	1174.	731.32	{308., 1393.}	74.0681	0.519282	newly 58 weeks cumul	6313.	831.53	{330., 1425.}	86.8283	1.12108
5	newly 3 weeks spec	228.	135.74	{23., 317.}	88.0702	0.623408	newly 3 weeks cumul	503.	711.25	{251., 1298.}	87.9026	0.25322	newly 5 weeks spec	460.	53.73	{3., 108.}	90.2978	1.41266	newly 5 weeks cumul	1330.	848.62	{313., 1796.}	81.5459	0.514131	newly 58 weeks cumul	6047.	949.18	{329., 2152.}	84.3033	1.02688
6	newly 3 weeks spec	208.	214.07	{5., 794.}	141.889	0.98643	newly 3 weeks cumul	401.	1355.83	{182., 6168.}	288.815	0.396643	newly 5 weeks spec	328.	40.26	{0., 67.}	93.811	2.01213	newly 5 weeks cumul	983.	1485.82	{182., 6718.}	160.242	0.588587	newly 59 weeks cumul	5568.	1555.6	{187., 7117.}	83.7076	1.06215
7	newly 3 weeks spec	360.	87.98	{5., 119.}	85.3389	1.00027	newly 3 weeks cumul	893.	568.05	{28., 573.}	79.1993	0.570217	newly 5 weeks spec	397.	31.95	{2., 65.}	92.5567	1.56958	newly 5 weeks cumul	1675.	651.12	{36., 704.}	82.4561	0.756337	newly 58 weeks cumul	4740.	717.64	{41., 796.}	87.3456	1.12386
8	newly 3 weeks spec	247.	97.37	{14., 158.}	72.3603	0.629838	newly 3 weeks cumul	499.	2082.9	{926., 6043.}	322.212	0.519852	newly 5 weeks spec	477.	16.18	{0., 22.}	96.608	2.30301	newly 5 weeks cumul	1346.	2133.87	{939., 6051.}	87.2117	0.246087	newly 62 weeks cumul	5812.	2152.35	{949., 6051.}	65.4283	0.578233
9	newly 3 weeks spec	71.	917.02	{33., 2110.}	1207.27	1.08153	newly 3 weeks cumul	127.	5084.3	{1316., 10 707.}	3910.02	1.52568	newly 5 weeks spec	311.	430.93	{1., 989.}	96.9743	0.728385	newly 5 weeks cumul	564.	6136.	{2122., 11 202.}	995.259	1.01509	newly 66 weeks cumul	6105.	7289.9	{2986., 12 444.}	50.7296	0.280771
10	newly 3 weeks spec	214.	988.93	{37., 2202.}	387.098	0.710508	newly 3 weeks cumul	335.	4698.06	{894., 9549.}	1314.42	1.07539	newly 5 weeks spec	555.	507.44	{2., 1093.}	62.764	0.67039	newly 5 weeks cumul	1279.	5909.26	{1428., 11 055.}	376.017	0.682483	newly 66 weeks cumul	6231.	7483.81	{2500., 11 476.}	54.0342	0.294434
11	newly 3 weeks spec	107.	730.69	{26., 1626.}	613.056	0.792686	newly 3 weeks cumul	156.	5664.82	{2251., 10 784.}	3533.29	1.46409	newly 5 weeks spec	375.	216.34	{0., 540.}	78.688	1.09763	newly 5 weeks cumul	750.	6273.77	{2556., 11 596.}	742.252	0.869199	newly 65 weeks cumul	5214.	6595.08	{2605., 12 413.}	58.2209	0.252126
12	newly 3 weeks spec	89.	690.5	{26., 1680.}	703.169	0.789747	newly 3 weeks cumul	220.	5567.35	{1431., 10 062.}	2436.17	1.29052	newly 5 weeks spec	266.	200.81	{0., 554.}	85.4774	1.04113	newly 5 weeks cumul	648.	6132.99	{1630., 11 539.}	856.529	0.91776	newly 65 weeks cumul	5759.	6428.95	{1630., 12 430.}	53.7401	0.288217
13	newly 3 weeks spec	161.	150.81	{18., 179.}	80.2671	0.403834	newly 3 weeks cumul	272.	2464.06	{916., 6479.}	808.963	0.801243	newly 5 weeks spec	444.	35.89	{0., 38.}	96.6824	1.99255	newly 5 weeks cumul	1004.	2568.84	{928., 6497.}	165.406	0.302466	newly 62 weeks cumul	6792.	2623.21	{934., 6499.}	69.9109	0.617537
14	newly 3 weeks spec	177.	600.32	{93., 1595.}	258.181	0.481263	newly 3 weeks cumul	299.	3730.93	{1172., 9300.}	1152.95	0.963259	newly 5 weeks spec	540.	170.32	{9., 377.}	70.1222	0.844407	newly 5 weeks cumul	1231.	4206.92	{1308., 10 545.}	253.215	0.481551	newly 64 weeks cumul	5655.	4468.52	{1314., 10 753.}	56.1556	0.355117
15	newly 3 weeks spec	125.	477.8	{10., 1305.}	323.584	0.682495	newly 3 weeks cumul	202.	3656.44	{455., 9015.}	1716.34	1.03455	newly 5 weeks spec	451.	324.13	{0., 994.}	97.7982	1.48598	newly 5 weeks cumul	842.	4361.77	{557., 9042.}	435.215	0.639678	newly 68 weeks cumul	6627.	5583.5	{593., 9801.}	50.1126	0.397809
16	newly 3 weeks spec	329.	582.01	{69., 1594.}	114.106	0.368924	newly 3 weeks cumul	674.	4051.46	{710., 9268.}	513.08	0.68812	newly 5 weeks spec	678.	155.2	{7., 366.}	79.2861	1.005	newly 5 weeks cumul	1835.	4491.68	{874., 10 492.}	167.105	0.414266	newly 64 weeks cumul	6828.	4745.72	{1010., 10 702.}	55.8448	0.413234
17	newly 3 weeks spec	187.	599.47	{110., 1559.}	240.872	0.449687	newly 3 weeks cumul	387.	4126.76	{1163., 9614.}	970.863	0.883571	newly 5 weeks spec	326.	163.37	{9., 365.}	69.0399	0.740503	newly 5 weeks cumul	968.	4590.17	{1338., 10 882.}	382.788	0.580561	newly 64 weeks cumul	6543.	4843.92	{1378., 11 658.}	59.8939	0.398771
18	newly 3 weeks spec	201.	115.92	{15., 224.}	70.5274	0.53752	newly 3 weeks cumul	460.	2244.57	{930., 6081.}	390.972	0.567978	newly 5 weeks spec	415.	14.87	{0., 29.}	96.4169	2.18924	newly 5 weeks cumul	1164.	2299.12	{945., 6090.}	112.995	0.254072	newly 62 weeks cumul	6707.	2311.38	{945., 6090.}	67.4567	0.608111
19	newly 3 weeks spec	266.	141.74	{12., 535.}	89.188	0.840117	newly 3 weeks cumul	588.	813.35	{54., 2073.}	110.481	0.457696	newly 5 weeks spec	499.	53.72	{1., 146.}	89.3226	1.58723	newly 5 weeks cumul	1512.	952.72	{75., 2806.}	88.6032	0.696435	newly 58 weeks cumul	6578.	1086.39	{98., 4571.}	84.6055	1.17638
20	newly 3 weeks spec	163.	153.41	{20., 561.}	104.62	0.587258	newly 3 weeks cumul	399.	875.63	{241., 3306.}	155.206	0.334911	newly 5 weeks spec	300.	53.66	{1., 143.}	87.4533	1.32848	newly 5 weeks cumul	931.	1020.44	{293., 4536.}	104.234	0.481443	newly 58 weeks cumul	5881.	1110.29	{316., 4741.}	82.6555	1.05289
21	newly 3 weeks spec	178.	936.4	{75., 2164.}	444.517	0.722328	newly 3 weeks cumul	310.	4451.52	{1305., 9316.}	1343.88	1.07479	newly 5 weeks spec	488.	480.14	{1., 1016.}	66.1803	0.660552	newly 5 weeks cumul	1116.	5593.63	{1947., 10 550.}	410.933	0.688702	newly 66 weeks cumul	6367.	7071.32	{2375., 11 187.}	49.5596	0.281417
22	newly 3 weeks spec	136.	578.21	{105., 1587.}	339.919	0.556199	newly 3 weeks cumul	315.	4076.35	{750., 9322.}	1199.44	0.970037	newly 5 weeks spec	337.	154.27	{9., 379.}	66.4955	0.7602	newly 5 weeks cumul	870.	4517.2	{984., 10 482.}	429.795	0.641796	newly 64 weeks cumul	5004.	4767.06	{1112., 10 681.}	60.1275	0.346226
23	newly 3 weeks spec	247.	322.85	{0., 793.}	131.502	1.18448	newly 3 weeks cumul	543.	4010.87	{737., 10 025.}	641.534	0.728013	newly 5 weeks spec	429.	97.42	{0., 311.}	88.3357	2.06489	newly 5 weeks cumul	1291.	4277.03	{759., 10 762.}	246.29	0.460649	newly 63 weeks cumul	5813.	4440.15	{762., 11 748.}	61.9974	0.391507
24	newly 3 weeks spec	98.	632.95	{103., 1595.}	551.867	0.679052	newly 3 weeks cumul	155.	3954.22	{1179., 9327.}	2453.55	1.26635	newly 5 weeks spec	358.	169.18	{11., 345.}	62.514	0.667381	newly 5 weeks cumul	700.	4440.9	{1348., 10 542.}	540.789	0.714421	newly 64 weeks cumul	6876.	4699.72	{1351., 10 750.}	56.3138	0.394468
25	newly 3 weeks spec	16.	973.28	{35., 1988.}	5988.25	1.4981	newly 3 weeks cumul	53.	4605.74	{1116., 8672.}	8590.3	1.80426	newly 5 weeks spec	207.	511.81	{0., 1238.}	207.319	1.02626	newly 5 weeks cumul	318.	5838.15	{1337., 9703.}	1739.62	1.17159	newly 67 weeks cumul	5792.	6974.34	{1439., 10 781.}	52.4327	0.274587
26	newly 3 weeks spec	172.	913.1	{27., 2086.}	458.837	0.841858	newly 3 weeks cumul	282.	4816.66	{461., 9863.}	1621.45	1.18937	newly 5 weeks spec	397.	445.09	{1., 1022.}	75.7154	0.702146	newly 5 weeks cumul	956.	5899.86	{1400., 11 209.}	532.477	0.827153	newly 66 weeks cumul	5226.	7173.24	{2856., 12 448.}	62.2047	0.318352
27	newly 3 weeks spec	68.	909.66	{31., 1974.}	1257.29	0.986796	newly 3 weeks cumul	149.	4821.79	{1135., 9528.}	3137.03	1.4016	newly 5 weeks spec	292.	481.2	{0., 1154.}	131.863	1.03672	newly 5 weeks cumul	586.	5973.55	{1444., 9744.}	921.029	0.929301	newly 67 weeks cumul	6353.	7047.29	{1524., 10 780.}	45.3158	0.225599
28	newly 3 weeks spec	54.	921.03	{40., 2116.}	1619.35	1.12293	newly 3 weeks cumul	104.	4825.88	{1324., 9841.}	4546.21	1.56618	newly 5 weeks spec	285.	432.82	{0., 994.}	106.961	0.75287	newly 5 weeks cumul	567.	5887.41	{1520., 11 102.}	945.952	0.993889	newly 66 weeks cumul	5472.	7068.29	{2976., 11 419.}	58.782	0.28486
29	newly 3 weeks spec	78.	456.85	{19., 1098.}	514.013	0.695886	newly 3 weeks cumul	105.	3839.18	{458., 9153.}	3560.88	1.31247	newly 5 weeks spec	382.	296.39	{0., 1023.}	108.081	1.51083	newly 5 weeks cumul	647.	4488.14	{561., 9174.}	605.855	0.719753	newly 68 weeks cumul	5977.	5539.21	{595., 9773.}	56.9883	0.419788
30	newly 3 weeks spec	42.	464.48	{16., 1121.}	1024.9	0.902934	newly 3 weeks cumul	59.	3913.56	{471., 9145.}	6536.61	1.56459	newly 5 weeks spec	274.	301.81	{0., 1028.}	130.252	1.42023	newly 5 weeks cumul	436.	4572.19	{583., 9149.}	957.589	0.879083	newly 68 weeks cumul	6478.	5624.31	{628., 9683.}	51.006	0.404817
31	newly 3 weeks spec	233.	662.04	{36., 1246.}	222.609	0.552299	newly 3 weeks cumul	480.	6204.61	{1952., 10 921.}	1193.85	1.02938	newly 5 weeks spec	432.	151.78	{0., 429.}	71.7037	1.20262	newly 5 weeks cumul	1241.	6664.48	{2058., 12 158.}	440.102	0.668069	newly 65 weeks cumul	5273.	6825.39	{2071., 12 408.}	59.1718	0.234307
32	newly 3 weeks spec	278.	123.72	{13., 181.}	86.9065	0.774198	newly 3 weeks cumul	694.	678.79	{46., 1270.}	88.0965	0.460312	newly 5 weeks spec	361.	46.75	{2., 81.}	88.1302	1.36443	newly 5 weeks cumul	1404.	800.87	{65., 1360.}	84.5249	0.643661	newly 58 weeks cumul	4849.	896.88	{72., 1372.}	84.4541	1.04733
33	newly 3 weeks spec	255.	128.07	{18., 318.}	82.3176	0.768418	newly 3 weeks cumul	589.	723.78	{76., 1181.}	91.5076	0.403224	newly 5 weeks spec	409.	51.44	{1., 103.}	87.9218	1.389	newly 5 weeks cumul	1337.	852.18	{99., 1647.}	82.899	0.615247	newly 58 weeks cumul	5959.	990.08	{134., 2555.}	84.8219	1.11627
34	newly 3 weeks spec	330.	109.67	{4., 153.}	84.5485	0.957723	newly 3 weeks cumul	818.	632.76	{31., 759.}	85.4303	0.574811	newly 5 weeks spec	455.	41.04	{2., 75.}	90.9802	1.50811	newly 5 weeks cumul	1672.	740.78	{39., 974.}	83.9282	0.767678	newly 58 weeks cumul	6027.	823.66	{48., 1175.}	87.7053	1.21673
35	newly 3 weeks spec	124.	704.3	{25., 1677.}	501.016	0.731885	newly 3 weeks cumul	254.	5565.88	{1645., 9975.}	2098.11	1.25384	newly 5 weeks spec	365.	187.79	{0., 546.}	80.8849	1.17186	newly 5 weeks cumul	843.	6105.82	{1650., 11 427.}	634.323	0.82825	newly 65 weeks cumul	5774.	6373.83	{1650., 11 737.}	53.3833	0.295879
36	newly 3 weeks spec	206.	24.5	{0., 67.}	88.5534	1.62377	newly 3 weeks cumul	457.	582.95	{172., 1066.}	73.512	0.281965	newly 5 weeks spec	363.	6.89	{0., 13.}	98.1019	2.65369	newly 5 weeks cumul	1101.	601.35	{187., 1102.}	60.4877	0.437812	newly 61 weeks cumul	6168.	612.21	{188., 1102.}	90.0744	1.13631
37	newly 3 weeks spec	223.	582.33	{100., 1550.}	186.175	0.400506	newly 3 weeks cumul	420.	4089.94	{1174., 9620.}	876.486	0.841081	newly 5 weeks spec	351.	148.89	{9., 367.}	68.7379	0.778261	newly 5 weeks cumul	1055.	4519.83	{1245., 10 868.}	334.649	0.523688	newly 64 weeks cumul	5466.	4734.26	{1296., 11 357.}	61.5909	0.351965
38	newly 3 weeks spec	171.	583.94	{101., 1524.}	257.181	0.453121	newly 3 weeks cumul	342.	4089.43	{1173., 9554.}	1096.81	0.923512	newly 5 weeks spec	402.	149.24	{9., 342.}	70.6816	0.795744	newly 5 weeks cumul	991.	4521.12	{1195., 10 816.}	361.748	0.54696	newly 64 weeks cumul	6742.	4734.65	{1255., 11 352.}	58.7628	0.392748
39	newly 3 weeks spec	270.	205.15	{2., 352.}	131.196	1.26638	newly 3 weeks cumul	603.	671.82	{19., 2185.}	126.119	0.693207	newly 5 weeks spec	373.	82.	{0., 205.}	97.2011	2.12642	newly 5 weeks cumul	1298.	901.23	{19., 2289.}	108.213	0.899423	newly 60 weeks cumul	6183.	1015.85	{19., 2322.}	88.2276	1.42393
40	newly 3 weeks spec	213.	293.33	{4., 653.}	178.746	1.03271	newly 3 weeks cumul	503.	745.07	{118., 2149.}	143.036	0.547912	newly 5 weeks spec	419.	142.32	{0., 818.}	99.6897	1.91788	newly 5 weeks cumul	1236.	1116.14	{118., 3390.}	121.338	0.796977	newly 60 weeks cumul	6409.	1392.48	{118., 6887.}	83.7931	1.29233
41	newly 3 weeks spec	69.	466.02	{10., 1086.}	604.203	0.765906	newly 3 weeks cumul	109.	3731.91	{467., 9024.}	3328.41	1.29077	newly 5 weeks spec	305.	313.95	{0., 1036.}	127.426	1.52176	newly 5 weeks cumul	561.	4414.52	{563., 9051.}	697.262	0.763583	newly 68 weeks cumul	6138.	5552.11	{635., 9766.}	55.7431	0.418124
42	newly 3 weeks spec	203.	187.07	{3., 760.}	136.606	1.00171	newly 3 weeks cumul	358.	1167.67	{156., 4710.}	282.249	0.403577	newly 5 weeks spec	454.	39.43	{0., 55.}	93.8833	2.10216	newly 5 weeks cumul	1061.	1287.57	{172., 5308.}	144.923	0.666526	newly 59 weeks cumul	6484.	1367.02	{176., 6769.}	85.6971	1.21339
43	newly 3 weeks spec	127.	910.12	{44., 1912.}	642.882	0.807057	newly 3 weeks cumul	210.	4691.25	{1142., 9101.}	2135.01	1.24074	newly 5 weeks spec	451.	486.92	{0., 1164.}	84.3548	1.01632	newly 5 weeks cumul	888.	5851.65	{1401., 9646.}	562.762	0.753793	newly 67 weeks cumul	6305.	6973.9	{1553., 10 546.}	43.8941	0.225469
44	newly 3 weeks spec	233.	28.8	{0., 73.}	88.	1.58391	newly 3 weeks cumul	482.	509.26	{149., 981.}	61.7469	0.309915	newly 5 weeks spec	403.	7.02	{0., 14.}	98.2581	2.585	newly 5 weeks cumul	1184.	528.32	{161., 981.}	61.8243	0.51378	newly 61 weeks cumul	5552.	538.06	{172., 981.}	90.3087	1.15319
45	newly 3 weeks spec	215.	640.41	{96., 1987.}	223.344	0.448474	newly 3 weeks cumul	394.	4086.96	{1056., 9315.}	943.34	0.877065	newly 5 weeks spec	444.	167.99	{8., 373.}	67.4482	0.809037	newly 5 weeks cumul	1183.	4568.07	{1120., 10 541.}	297.304	0.512152	newly 64 weeks cumul	6681.	4820.61	{1245., 10 748.}	57.5472	0.400338
46	newly 3 weeks spec	191.	282.3	{0., 722.}	135.131	1.0552	newly 3 weeks cumul	452.	3691.16	{733., 8867.}	720.894	0.77562	newly 5 weeks spec	337.	84.94	{0., 277.}	85.0445	1.82387	newly 5 weeks cumul	1052.	3921.27	{757., 10 196.}	284.493	0.498648	newly 63 weeks cumul	5089.	4102.46	{760., 10 196.}	60.8803	0.373805
47	newly 3 weeks spec	210.	676.44	{102., 1996.}	243.362	0.483946	newly 3 weeks cumul	472.	4260.66	{1188., 9365.}	808.814	0.833513	newly 5 weeks spec	303.	186.23	{9., 435.}	66.8218	0.671659	newly 5 weeks cumul	1066.	4788.92	{1332., 10 895.}	358.923	0.579498	newly 64 weeks cumul	5261.	5082.72	{1361., 11 367.}	61.6845	0.348224
48	newly 3 weeks spec	153.	28.01	{0., 64.}	84.268	1.37454	newly 3 weeks cumul	281.	574.18	{151., 926.}	135.637	0.340907	newly 5 weeks spec	442.	6.34	{0., 10.}	98.5656	2.71377	newly 5 weeks cumul	972.	593.75	{162., 1139.}	62.822	0.436625	newly 61 weeks cumul	6845.	604.06	{164., 1285.}	91.1752	1.21565
49	newly 3 weeks spec	191.	329.89	{0., 797.}	163.22	1.14538	newly 3 weeks cumul	421.	3895.29	{740., 9468.}	827.109	0.810042	newly 5 weeks spec	403.	98.73	{0., 289.}	88.1067	1.90516	newly 5 weeks cumul	1126.	4167.41	{760., 10 783.}	284.626	0.492953	newly 63 weeks cumul	5314.	4335.73	{761., 11 800.}	63.7006	0.385958
50	newly 3 weeks spec	165.	493.16	{6., 1235.}	248.885	0.660705	newly 3 weeks cumul	248.	3602.57	{463., 8951.}	1361.03	0.958538	newly 5 weeks spec	521.	332.62	{0., 1013.}	92.833	1.54365	newly 5 weeks cumul	1082.	4331.66	{571., 8972.}	325.366	0.582119	newly 68 weeks cumul	6795.	5496.59	{587., 9785.}	50.147	0.41859
